# Reflections on the Cloak of Convenience

**DOI:** 10.1007/s11673-025-10449-0

**Published:** 2025-06-10

**Authors:** E. Felman, I. Kerridge, M. Vered, P. Komesaroff

**Affiliations:** 1 Monash University, Melbourne, VIC Australia; 2 The University of Sydney, Sydney, NSW Australia

**Keywords:** Digital technologies, Artificial intelligence, Ethics

## Abstract

A key maxim guiding the introduction of new technologies, including those utilizing artificial intelligence, is that such technologies should carry rewards of “convenience”: indeed, the more “convenient” a new technology is considered to be, the more likely it is to be welcomed and adopted. Rudimentary examples from last century include the microwave, washing machine, and dishwasher; more recent innovations from the present century include portable navigation systems, online shopping applications, internet search engines, smart phones, telehealth, automated workplace systems and processes, email and messaging technologies, and—most recently—large language models that are able to undertake multiple complex tasks. Each of these technologies offers a variety of benefits. However, a unifying feature is that all have been considered to enhance convenience, understood as saving time and/or effort. In this paper we explore the provenance and meaning of the—usually unexamined—concept of convenience, identifying an unexpected link with erosion of values and depletion of the diversity and richness of personal experiences. We conclude that the prioritization of convenience as a driver of innovation carries with it risks, which may go unnoticed or be difficult to discern.

A key maxim guiding the introduction of new technologies, including those utilizing artificial intelligence, is that such technologies should carry rewards of “convenience”: indeed, the more “convenient” a new technology is considered to be, the more likely it is to be welcomed and adopted. Rudimentary examples from last century include the microwave, washing machine, and dishwasher; more recent innovations from the present century include portable navigation systems, online shopping applications, internet search engines, smart phones, telehealth, automated workplace systems and processes, email and messaging technologies, and—most recently—large language models that are able to undertake multiple complex tasks.

Each of these technologies offers a variety of benefits. However, a unifying feature is that all have been considered to enhance convenience, understood as saving time and/or effort. In this paper we explore the provenance and meaning of the—usually unexamined—concept of convenience, identifying an unexpected link with erosion of values and depletion of the diversity and richness of personal experiences. We conclude that the prioritization of convenience as a driver of innovation carries with it risks, which may go unnoticed or be difficult to discern.

We will first introduce the historical and theoretical context of convenience, including the notion of “convenience orientation,” as well as describing some of the key features of convenience. Following this, we will discuss the idea of expanding technological interconnectivity, usefully conceptualized as “the Internet of Things,” according to which the expansion of digital technologies, including AI, are assumed to enrich social and individual capacities. We then explore the unintended consequences associated with the deployment of convenience as a guiding objective for the development and implementation of digital technologies.

We argue that convenience is a quality that may save time and effort but often also comes with a price: the displacement or erosion of other beneficial qualities. Convenience therefore operates as a veil; it masks the disposal of positive traits to create an enticing iteration of a product or service with a time and/or effort saving. It sanitizes our interactions and risks reducing the quality and quantity of interpersonal relationships and human interactions, both in a conceptual and practical sense. There is therefore a need for the development of critical awareness of the risks and benefits associated with the prominent role commonly granted to convenience in relation to the assessment and regulation of new technologies.

## Convenience as a Characteristic

Awareness of convenience as an attribute that has value in its own right has been amplified by the widespread and fundamental use of the internet, which promotes connectivity as a primary rationale, and convenience as a secondary motive. The English word “convenience” originated in the late fourteenth century from the Latin word *convenientia* meaning “a meeting together, agreement, harmony.” It was commonly used as a term to reference “conformity, resemblance, similarity” which was then adapted into its contemporary meaning of “that which gives ease of comfort; a convenient article or appliance” in the 1670s (Online Etymology Dictionary 2024).

The idea of convenience as a beneficial quality of a product or service has a surprisingly recent and relatively superficial origin: it was first used as a positive attribute in 1923 as a service or goods descriptor to market categories of “low-effort” supplies (Brown 1990). Convenience as a more commonplace descriptor of a group of products or services has an even more recent origin, beginning in 1965 (Online Etymology Dictionary 2024). Use of the term “convenience” in its colloquial sense started as a marketing strategy designed to appeal to consumers to sell “quick” goods or services. Its popularity has since proliferated into many products and services which have been developed and produced with the aim of saving customers time and effort. However, despite its striking success in the world of products and consumers, we know that the qualities of convenience do not necessarily have meritorious underpinnings, such as safety, reliability, and quality. Nevertheless, convenience is now considered valuable in a time-poor, service-driven society.

Convenient products were defined as “a reduction in the amount of consumer time and/or energy required to acquire, use, and dispose of a product or service relative to the time and energy required by other offerings in the product-service class” (Online Etymology Dictionary 2024). Some forty years later, this type of product then developed into an entire category, and later industry, known as “convenience goods” (Bagozzi and Dholakia 1999). In early marking usage, convenience denoted the time and effort consumers used in purchasing a product rather than a characteristic or attribute (Brown and McEnally 1992).

Preliminary marketing research on the convenience construct examines factors which increase the demand for convenience. These include the employment status of the adult members of the household—as the wife or second earner enters the workforce, there is less time available to perform tasks around the home and consequently an increased demand for convenient products or services (Brown 1990). Socioeconomic status is another factor which has been indirectly linked to convenience consumption—role overload such as maintaining dual responsibilities in the home and employment is a strong discriminator of convenience goods orientation (Reilly 1982). The scarcity of time caused by changes in lifestyle also drives demand for convenience. Consumers may “time-buy” by investing in products and services that reduce time expenditure (Graham 1981).

Another factor which may produce an orientation towards or away from convenience is consumer values. For example, customers prioritizing carbon footprint or environmental values may only pursue convenience when it is cost-effective *and* environmentally supportive (Stampfl 1982). Domain-specific values differentiate cost-orientation and convenience-orientation. The convenience-oriented consumer seeks to accomplish a task in the shortest time with the least expenditure of human energy whereas the cost-oriented consumer bases selection on minimizing financial expenditure. Research has shown that the primary distinguishing variables between the two groups are age, household income, and family-type (Morganosky 1986). The level of demand for convenience is also affected by the product type, with convenience food, clothing, and household products appealing to customers in different ways (Stampfl 1982).

It can be difficult to conceptualize convenience and examine its underlying elements. Early marketing research concentrated on why consumers want convenience rather than what convenience is. The decision-making process in selecting a product or service is multifactorial and seemingly not necessarily straightforward (Stampfl 1982):

In the face of time and energy poverty, the consumer must decide if the price of an offering is appropriate given the time and energy required or the time and energy saved by the product. Thus, an offering requires a certain amount of time and energy to acquire, use and dispose of it. The decision to prioritise convenience by selection of a product or service occurs when: 1) the price of that offering is appropriate to the required expenditure of time and energy relative to the other options the consumer faces, 2) the consumer sees the offering as producing (through time and energy savings) time and energy that can be used for other purposes, or 3) the offering allows the consumer to accomplish something that is not possible, given the consumer’s time and energy budgets, without the product or service.

### The Example of Home Cooking

It is important to note that this difference in time and effort might seem trivial but such a change in priorities can have significant and widespread consequences. An interesting example to demonstrate the impact of convenience is the evolution of home cooking. For centuries, home-cooking was traditionally undertaken by a housewife who usually did not seek paid external employment so that she could maintain a home, care for children, and provide home-cooked meals for her family. However, cooking at home is time and labour intensive; at a minimum, the task requires meal planning, food preparation skills, access to kitchen equipment, time and money to shop for ingredients, time to transform a collection of food ingredients into a meal, and time and effort involved in cleaning up afterwards.

The American TV-dinner first appeared in 1954 as a time and effort saving product (Brown 1990). This development was innovative by providing quicker and easier meal options in the home during an era when eating out and take-away outlets were sparser than today. TV-dinners were popular globally, and by 1959 the definition of convenience foods was extended to include “products of the food industries in which the degree of culinary preparation has been carried out to an advanced stage and which are purchased as labour-saving versions of less highly processed products” (Ridgwell 1996, 116). The establishment of TV-dinners to eat in the home was revolutionary—its introduction and aim was distinct from other convenient dining options such as eating in a restaurant, café, canteen, or drive-through, which all took place outside the home.

The trajectory of prioritizing the qualities of convenience in home cooking continued for decades. The introduction of TV-dinners was followed by an influx of domestic technology devices such as microwave ovens in the late 1980s (Jabs and Devine 2006), and to a lesser extent bread machines, vacuum sealers, and ice-cream-makers designed and sold for use at home. Many causes may explain the growing trend of convenience foods and devices, including the changing household structure, female participation in the labour force, inventive manufacturers, appealing advertisement, ownership of kitchen technology, individualism, time usage, and declining cooking skills (Scholliers 2015).

Traditional home cooking is still important and valuable, in that it provides greater benefit than any of the convenient alternatives such as home delivery, pre-packed ready-made meals, and takeaway. Home cooking has a positive influence on health and nutrition (Stead, et al. 2004), increases vegetable and fruit consumption (Wrieden, et al. 2007), improves accessibility of fresh produce at home (Michaud, Condrasky, and Griffin 2007), and increases the likelihood of meeting dietary guidelines for fruit, calcium, and vegetable consumption (Larson, et al. 2006). The experience of home cooking is also superior in other, less obvious, ways. Home cooking gives families control over their food supply, helps them to connect with others, enables them to explore their own food culture, and allows teenage members of the family to become more independent (Simmons and Chapman 2012). These demonstrated advantages are not transferred or even transferrable to the more convenient home-cooking options.

Over the past seventy years, home cooking has been transformed dramatically from its traditional meaning to even more convenient iterations. The change is so striking that the original idea of home cooking has now burgeoned into an entire industry of eating food in the home which has been prepared externally. Options include home delivery, take away, third party rideshare services, meal boxes, and frozen meals. The products and services undoubtedly save time and reduce effort, but they are so far removed from the original idea of home cooking that they can no longer be considered home cooking at all! Indeed, the benefits are *only* the saving of time and effort—all other established qualitative advantages to home cooking have been abandoned and eliminated to preserve the value of convenience.

Within a matter of decades, convenient options of home cooking have evolved with a priority of time and effort savings in this domain. Convenience in terms of food is now linked to a range of options involving decision, access, transaction, and benefit such as convenience shopping, convenience storing, convenience cooking, convenience eating, and convenience cleaning (Jaeger and Meiselman 2004). The allure of convenience is so popular and desirable, that it erases the benefits of the original iteration (in this example, home cooking) and creates an illusion of a “better” product or service (such as a pre-prepared meal which is re-heated and eaten in the home). The discarded benefits are so well-masked, that at times it is almost impossible to determine their original content. This then leads to the fundamental question–- what are the positive aspects of an experience which are being reduced or even sacrificed for the sake of augmented time and effort improvements?

## Elements of Convenience

The trade-off between effort and quality continues today in many domains. The notion of a convenient solution often requires a sacrifice of constitution in exchange for the priority of resources such as temporal, spatial, and effort dimensions. Marketers developed the concept of convenience as an attribute that reduces the non-monetary price of a product (Gehrt and Yale 1993). The issue of non-monetary cost is central to the concept of convenience, and the existing literature relating to time and effort is particularly relevant here.

### Time

Time is a limited and scarce resource, and unlike money it cannot be expanded and is finite. Objective time is a continuous metric which can be measured by a clock or timer—for example, the actual minutes which pass by while a patient is waiting for an appointment as measured by a clock. Subjective time is based on perceptions and influenced by psychological factors, such as an individual’s perspective on waiting room delays based on their thoughts and feelings during that time (Hornik 1984). “Saving time” refers to the idea of reallocating time across activities to increase efficiency or to allot less time for unenjoyable or low priority endeavours. Even though time usage can be perceived as either an investment or cost, it is more commonly considered an expenditure.

Rational choice is an economic theory which suggests that individuals use rational calculations to make rational choices and achieve outcomes that are aligned with their own personal objectives, which maximize an individual’s self-interest (Scott 2000). The use of rational choice theory predicts outcomes that provide the greatest benefit and satisfaction in the context of limited available options. In this model, time, like income and price, constrains choice. Therefore, from a consumer perspective, time is sold in the labour market and bought with time-saving goods and services; this is known as a time budget allocation approach (Graham 1981). Such an approach views the cost of time as relinquished income or a lost opportunity to participate in other preferred activities. The origins of convenience as a marketing concept assumes that there is a relationship between time scarcity and desire for goods and services that offer convenience (Berry, Seiders, and Grewal 2002).

Consumer behaviour research on time focuses on time allocation, temporal orientation and perception, and cultural influences. Time allocation is an outcome of demographic, socioeconomic, and psychological decisions, as well as lifestyle and consumption behaviour. Temporal orientation includes perceived time scarcity and sensitivity to time-related issues. Cultural differences can affect the evaluation of convenience and therefore the important of time differs internationally (Gehrt, Yale, and Lawson 1996).

Time has been classified by researchers according to work and non-work roles, with the latter including personal care, household maintenance, and leisure. These classifications allow an understanding of the significance of non-economic variables and explain the reason people may seek to prolong rather than minimize time expenditures in certain situations.

### Effort

“Effort” is related to time, thought, and emotion. Like time, it is a distinct non-monetary cost which influences perceived convenience and satisfaction. Energy expenditure, in addition to time and money constraints, influences consumer decision-making (Berry, Seiders, and Grewal 2002). Generally, the more effort a person exerts, the greater the expected outcome in return.

Existing research almost exclusively focuses on time rather than effort and perceives and analyses effort-saving as time-saving, even though there is a distinction. Physical effort has received little attention in consumer research, and cognitive and emotional effort has not been widely studied (Wales 2009). Research has shown that individuals are aware of their own cognitive resource limitations and therefore consciously conserve these resources during decision-making (Gehrt and Yale 1993). Studies suggest that we have a limited ability to estimate or predict the effort required by a task and that there are significant individual differences in perceptions of required effort (Brunner, Van der Horst, and Siegrist 2010).

### Convenience Orientation

“Convenience orientation” combines the time and effort saving considerations into a singular measurement—it refers to an individual’s general preference for convenient goods and services. Convenience orientation was first studied in 1971 in relation to the use of convenience-oriented food and appliances (Anderson 1971). Convenience orientation as a behaviour was found to have a significant impact on consumer decisions, as well as being related to a distinct consumption strategy of a consumer seeking to accomplish a task in the shortest time with the least expenditure of human energy. Convenience orientation is the fundamental value consumers place on goods and services with inherent time-saving or effort-saving characteristics, which has a significant impact on consumer decision-making (Morganosky 1986). Even though convenience orientation has been studied, the findings have been inconclusive and generally focused on goods rather than services, as well as the exploration of cost preferences versus convenience orientation (Voli 1998).

## Digital Technologies, Artificial Intelligence, and Final Reflections

The focus on convenience in relation to new digital technologies originated with the idea of the “Internet of Things” in around 1999 (Ashton 2009). The Internet of Things was conceived as utopian web of constant real-time connectivity between people and technology, with “people and things to be connected Anytime, Anyplace, with Anything and Anyone, ideally using Any path/network and Any service” (Sundmaeker, et al. 2010, 11). In theory, this could allow all people to have access to an Internet environment populated by self-managing smart technology anytime and anywhere for any purpose. Originally proposed as an objective of the internet in its earlier development, the Internet of Things has been expanded to technological and artificial intelligence development more broadly. It is now manifested with a variety of technologies to connect digital and physical domains. Physical objects are embedded with digital technologies such as sensors and actuators. These technologies communicate with computing system via wired or wireless networks. In this way, computers can monitor or manage the status and actions of connected objects and machines.

Some technologies which have been highlighted under this Internet of Things umbrella include seamless connection of devices, intelligent cars, machine to machine communication, smart buildings, things-to-person communication, and thing-to-thing communication (Nolin and Olson 2016). “Things” have been categorized as a hierarchy of five domains. At the lowest level, *domain 1*, there are real world entities or virtual entities that communicate with each other and with infrastructure. At *domain 2*, things can compete with other things regarding resources and services. They can be equipped with sensors and therefore interact with the environment. Higher up, at *domain 3*, they can communicate and collaborate with other things and create groups or networks. More power is given to things at *domain 4*, at which level they are considered autonomous. Here they can negotiate and adapt to their environment, they can also extract information and patterns from environment. They are expected to be able to learn, take decisions, and reason. At the highest level, *domain 5*, things are capable of self-replication, controlling, creating, managing, and even destroying other things (Scott 2000).

The Internet of Things is an “alpha convenience” proposition. This term means that convenience is essentially prioritized in every technological development and interaction which was originally considered to be the fundamental goal of technology. In a time and effort-driven society, perhaps the concept of alpha convenience is a seductive one. The prioritization of convenience is amplified further in the evolution from technology to digital technology, to now artificial intelligence whereby the generative element of the technology in inherently a further time and effort saving. Therefore, the arguments about convenience explored above apply even more so to artificial intelligence technologies. The examples of satellite navigation systems and automated social media content will illustrate this.

### The Example of Satellite Navigation Systems

Satellite navigation is based on a global network of satellites that transmit radio signals from medium earth orbit. The 31 Global Positioning Satellites (GPS) within this system have been developed and operated by the United States and provided to the international community free of charge. The GPS service provides location position with seven meter accuracy 95 per cent of the time anywhere on or near the earth’s surface. The use of GPS has allowed consumer mapping and navigation products to become widely used over the past fifteen years. Consumer satellite navigation devices provide users with their location and a route to a particular destination, essentially replacing conventional road maps requiring manual routing and destination locating. The GPS devices were originally stand-alone and portable and have now been integrated into phones, cars, and other devices.

There is no doubt that the GPS technology has provided a very convenient alternative to manual mapping, which is part of its allure for consumers. By providing an automated “ideal” route based on tolls, traffic, time, and (using generative artificial intelligence), the technology allows people to save time and effort which would otherwise be expended finding a manual destination and route on an analogue map. It seems that nothing is lost in this exchange—it is suggested that use of this technology saves time and effort, with no other values lost in the trade. However, this is not entirely true. There are values which are attenuated or, in some cases, lost altogether, such as navigational skills and appreciation of spatiality. Whether the use of the technology is worth this trade-off is for the user to decide, but it is important to understand and acknowledge the qualities which have been abandoned.

There are also values which are lost that are less obvious to the consumer. Elizabeth Grosz explores the “constitutive and mutually defining relations between bodies and cities” in her essay “Bodies-Cities” in *Space, Time and Perversion* (115). She proposes that the built environment of the city provides “context and coordinates for contemporary forms of body” resulting in a more complex relation between bodies and cities than traditionally realized. A fundamental quality of the city is its “unintegrated and ad hoc” nature which connects “a number of disparate social activities, processes, relationships with a number of architectural, geographical, civil and public relations” (Grosz 1995, 116). This “ad hoc” quality of cities is seemingly lost in the ordered algorithm of satellite navigation which demands that the consumer slavishly follow the designated route.

Michel De Certeau also connects the body and the city—his essay “Walking in the City” in *The Practice of Everyday Life* draws similarities between urban systems and language, with improvizational walking including shortcuts and wandering analogous to informal spontaneous language such as turns of phrase, inside jokes, or anecdotes. He states that “the act of walking is to the urban system what the speech act is to language or to the statements uttered” (97). He describes the city, and particularly the act of wandering, as a space of enunciation where walkers demonstrate possibilities through their walking choices. He states that “the walking of passers-by offers a series of turns and detours that can be compared to ‘turns of phrase’ or ‘stylistic figures.’ There is a rhetoric of walking” (de Certeau 1984, 100). Therefore, in eliminating this style of casual exploration using satellite navigation technology, there is a hidden impact upon and change to the body and sense of self.

### The Example of Automated Social Media Content

Another example of “convenient” generative artificial intelligence is automated social media content. Social media platforms perform a content analysis using software which identifies specific words, phrases, and topics. The words, phrases, and topics are linked to other related topics using an algorithm. This algorithm is then engaged for the social media platform’s benefit by providing targeted advertising and marketing. For example, a user on Facebook might search for COVID-19 treatments. Over the coming days, this user may see paid advertising from drug manufacturers, tissue companies, and work from home devices on the basis that the algorithm has linked a search of “COVID-19 treatments” to the purchase of medications, tissues, and working from home devices. This idea, which is essentially a “convenient” expansion of targeted advertising, is on one hand seemingly harmless but on the other can be insidiously manipulative and controlling.

From a benevolent perspective, the social media user may be allured by the time and effort saved with the advertised information. This is particularly the case should the algorithm accurately predict the related topics, and the advertised content is desired by the user.

A less optimistic angle might consider that automated content generated in this way negatively impacts on the social media user. Automated social media content discourages the user from searching for independent information and facilitates the spread of misinformation. It also influences the social media user by surreptitiously providing content designed to keep users scrolling (and spending money), rather than prioritizing facilitating access to accurate information. Automated content generated in this way also assumes that the information is desired or accurate, purely for the purposes of marketing and convenience. A further concern is that the algorithm generating automatic content is based on the search terms of the user. For example, searching “COVID-19 treatments” may be relatively innocuous, but searching “COVID-19 conspiracy theories” would result in the user being bombarded with other (false) conspiracy theories for a period. By relying upon this “convenient” generative artificial intelligence technology, a saving of time and effort results in a loss of other beneficial qualities such as freedom of information in an open platform and control of negative or false information.

The pervasiveness of social media in everyday life has impacted upon millions of individuals. Social media’s widespread reach, use, and communication channels facilitate and incentivize opportunities for abuse, particularly as social media activity has become increasingly intertwined with the events of the offline world. These platforms can be exploited to spread misinformation, deceive, and manipulate by auto-generating content which at best are not sought out by the user and at worst may not be relevant or desired. The lack of effective content verification has resulted in making social media useful and accessible on one hand and unsafe and unhealthy on the other. Automated information “controls” the user in a sense, by way of improper communication such as misinformation campaigns, bots, and spam resulting in fear and manipulation, particularly in vulnerable and impressionably populations such as younger social media users.

Connecting to others via social media avoids an in-person interaction—there is no need to travel to a common destination or even be available at the same time. Social media connections allow people to connect from different places and at different times thereby saving effort and time. However, most would acknowledge the losses of such an interaction such as reducing the meeting to a series of messages, not being able to meet the other person face-to-face, and not enjoying a real-time dynamic in-person interaction. Some might argue that this fundamentally dilutes the interaction to the extent that it is no longer an interaction at all. Others could claim that this saving of time and effort in using social media to interact allows additional time to undertake other activities such as reading, spending time with loved ones, or even creating a “hierarchy” of interactions whereby some less important (or familiar) interactions can be reduced to social media leaving more time for preferred interactions such as with close friends and family. While this is theoretically possible, or even probable, this is not the practical reality. Early internet research found that time spent online displaced time spent with friends and family (Kraut, et al. 1998), thereby compounding the negative impact of prioritizing convenient communication.

The internet has also had an impact on the quality of our social interactions. Many factors drive changes in the organization of social relationships. It has been suggested that industrialization and urbanization has caused the weakening of community and family bonds (Parigi and Henson 2014). New technologies can also change social relations. The internet allows people to keep in touch with large groups of friends, families, acquaintances, and even strangers all over the world. It substantially increases the quantity of communication with altered relationship dynamics and ways of maintaining relationships. It is widely accepted that a greater number of connections to people means that there is less attention given to each individual (Mayhew and Levinger 1976). Therefore, the accessibility of digital communication results in an abundance of virtual social interactions, with our social relationships becoming more superficial (Turkle 2011), and small groups of strong local ties have been substituted for a larger set of weak ties (Chen 2013).

Almost twenty years before the introduction of virtual communication, Christopher Lasch wrote about the development of “pathological narcissism” in society because of the decline of the family unit in American society (Lasch 1979). This problem has been amplified in the digital revolution—virtual channels of communication have been associated with rising individualism and linked to negative consequences for social relationships (Vriens and van Ingen 2018). The negative consequences of digital communications are not as a result of the volume of social interaction but rather the decline of strong ties and quality of relationships. Despite these negative consequences, the convenience of the mode of communication has masked these obviously lost values.

## Concluding Comments

The examples we have discussed (satellite navigation, auto-generated content on social media, and virtual communication) exemplify the unique way in which an emphasis on reducing time and effort may undermine other values by creating an artificial “vacancy” of time and effort for other activities and thereby masking the lost qualities. This raises the question of how it came to be that the role of convenience was extended so widely in the development and implementation of digital technologies and artificial intelligence.

Convenience as a recent practical objective has an explicit societal value associated with it, being the prioritization of time and effort saving. Technology and artificial intelligence offer convenience as a benefit, and this quality can potentially overshadow other benefits. The “overshadowing” or loss of other benefits is because of the fundamental saving of time and effort. By saving time and effort, we seem to forget or not care about other elements which may be lost. Perhaps this is human nature or the society’s predisposition to value certain (selfish?) qualities over others. Maybe, too, a saving of time means that this extra time gained can be filled with preferred activities.

Discussions in computer science literature treat convenience as unproblematic in the sense that it is always beneficial, meaning that if we can identify areas of human action where efficiency can be enhanced then the technology should be produced (Thompson 2005). However, given the mask of convenience explained earlier, are there limits to how convenient technology should become? What are the advantages which are side-lined to absolutely prioritize convenience? There are no definitive answers to these questions—consideration of these issues is dependent upon the technologies in question, the qualities sacrificed for the sake of convenience, and the preferences of the users of the technology. It is important to interrogate current societal values and remember that convenience is only one benefit of possibly many others in a new or existing technology. It is also important to try to determine the other lost values which may be masked behind the dazzle of (alpha) convenience.

In the home cooking example, some of the qualities lost (never to be re-gained) include social connection, health benefits, and the development of culinary skills. Similarly, using a technological example, the digitization of human interactions using phones, emails, and videocalls has created an easier and quicker way to connect. Paradoxically, by facilitating an easier method of communication with others, the technology itself has also created a distance between the users by reducing the richness and depth of an in-person human connection.

There is no clear solution to this trade-off, particularly as society develops within an ever-increasing time-poor digitally oriented world. It is for each individual to consider whether the predilection for convenience is worth the sacrifice of other benefits. This paper highlights the somewhat superficial origins of the concept of convenience (to sell products and services), explores the meaning of the term, and reiterates that there is a balance to be struck between time and effort savings and other vital societal values such as human connections and interpersonal interactions.
